# Cell based functional assays for IDO1 inhibitor screening and characterization

**DOI:** 10.18632/oncotarget.25720

**Published:** 2018-07-20

**Authors:** Thomas Richards, Elena Brin

**Affiliations:** ^1^ Polaris Pharmaceuticals, San Diego, CA, USA

**Keywords:** IDO1, cell-based assay, epacadostat, BMS-986205

## Abstract

Indoleamine 2,3-dioxygenase 1 (IDO1) is a new immune-oncology target and its inhibitors have shown promise in the clinic especially in combination with other immune-stimulating agents. Here we describe two robust cell-based assays for screening IDO1 inhibitors. Both assays can be easily adopted by most laboratories and utilized for screening of IDO1 inhibitors. Endogenous IDO1 expression is induced in a cancer cell line with interferon gamma and its activity is assessed by measuring kynurenine secreted into the media. The effect of cancer cell IDO1 induction and inhibition on T cell activation is evaluated in a co-culture assay using Jurkat T cell line. Additional readouts assessing cell viability are employed for early detection of false positive IDO1 inhibitors and toxic compounds. Clinical candidates epacadostat and BMS-986205 were evaluated in the assays as control compounds, the former can completely inhibit IDO1 activity while the maximum effect of the later is limited (to about 80% in our system) consistent with the differences in their interaction with IDO1. Nanomolar concentrations of both compounds rescued IDO1 mediated inhibition of T cell activation. However, treatment with micromolar concentrations of BMS-986205 blocked Jurkat T cell activation and after prolonged incubation induced cell death.

## INTRODUCTION

Indoleamine-2,3-dioxygenase (IDO or IDO1) is an intracellular monomeric, heme-containing enzyme catalyzing the conversion of L-tryptophan (L-Trp) to N-formylkynurenine - the first and rate-limiting step of the L-Trp catabolic pathway [1]. IDO1 was found to be broadly expressed in human tumors [2–4] and thought to contribute to cancer development primarily by facilitating tumor immune escape [5–8]. T cells are very sensitive to low tryptophan levels and can undergo cell cycle arrest and cell death under tryptophan deprivation conditions [9, 10]. Low tryptophan and kynurenine metabolites induce effector T cell anergy, decrease tumor immune cell infiltration and increase regulatory to effector T cell ratio [3, 10, 11]. In addition to cancer, IDO1 plays role in autoimmune disorders, immunosuppression and chronic infection [7].

Several IDO1 inhibitors are being evaluated in clinical trials for various cancer indications as monotherapy and in combination with immune therapies (e.g. checkpoint inhibitors) or chemotherapy [12]. Additional efforts are underway to discover novel IDO1 inhibitors.

IDO1 can be activated by many stimuli. Interferon gamma (IFNγ) and tumor necrosis factor alpha (TNFα) are the strongest inducers of IDO1 [6, 13, 14]. IDO1 genes have IFN response elements and are induced at local inflammation sites in several cell types including dendritic cells, macrophages, eosinophils, epithelial and endothelial cells [15]. Here we used an ovarian cancer cell line SKOV-3 that endogenously expresses IDO1 upon IFNγ treatment to develop functional assays for screening IDO1 inhibitors.

To mimic the effect of IDO1 on T cell activation in a tumor microenvironment we utilized a co-culture of IDO1 expressing SKOV-3 cells with Jurkat T cells. Jurkat is a human leukemic T cell line that secretes IL-2 when stimulated by mitogens or superantigens [16].

Two compounds that are currently being evaluated in clinical trials, epacadostat (Incyte) and BMS-986205 (Bristol-Myers Squibb), were tested in the assays as exemplary IDO1 inhibitors and can be used as assay controls. Cell-based assays compliment enzymatic assays by interrogating the ability of compound(s) to cross cell membranes without causing cytotoxicity and inhibit IDO1 in a cellular context.

## RESULTS AND DISCUSSION

### SKOV-3 cell-based IDO1/Kynurenine assay

SKOV-3 ovarian cancer cell line over-expresses IDO1 and its expression is further increased in response to IFNγ treatment [17]. We used this cell line as a basis of the two assays inducing IDO1 with IFNγ to enable compound evaluation for inhibition of IDO1 function. The cells that have not been exposed to IFNγ served as a negative control. SKOV-3 cells have been described to express IDO1 even in the absence of IFNγ [17]. However, under the assay conditions described below we detect IDO1 activity only after IFNγ exposure. Perhaps, the difference in incubation time (24 h versus 72 h) and/or differences in source or culturing of the cells are responsible for this discrepancy.

Cell plating and IDO1 induction was conducted as described in Materials and Methods.

On the day of the assay, two days after the cell plating, test compounds were serially diluted in freshly prepared Assay Medium (McCoy's 5a, 10% FBS, 2 mM glutamine, 50 μg/mL (244.822 μM) L-tryptophan). The cell culture medium from SKOV-3 containing plate(s) is then replaced with 200 μL of Assay Medium containing test compound or vehicle control. After compound addition cells were incubated for 24 h at 37°C in a 5% CO_2_ incubator.

The next day 140 μL of conditioned medium was removed from each well of the cell culture assay plate(s) and transferred into a fresh 96-well plate(s) to be analyzed for N-formylkynurenine content as described by Feng and Taylor [18] and Yue *et al*. [19].

Briefly, 10 μL of 6.1 N trichloroacetic acid was added to each well and the plate was incubated at 50°C for 30 min to hydrolyze N-formylkynurenine into kynurenine. The sediment was then removed by centrifugation at 2500 rpm for 10 minutes and 100 μL of supernatant was transferred into transparent 96-well plate and mixed with equal volume (100 μL) of freshly prepared Ehrlich's reagent. After 10-minute incubation at room temperature absorbance was read at 480 nm using a microplate reader.

Under the assay conditions all media tryptophan is converted into kynurenine as we detect approximately 50 μg/mL of kynurenine in the media of non-treated control cell wells (same concentration as tryptophan added to the assay media). If SKOV-3 cells are seeded at 1 × 10^4^ cells/well only half of the tryptophan in the media is converted into kynurenine (data not shown).

Kynurenine detected in the test wells was expressed as percentage of that in non-treated control wells (~50 μg/mL) and plotted against compound concentration (log transformed). Four parameter logistic nonlinear regression model was used for curve-fitting analyses.

**Figure 1:**
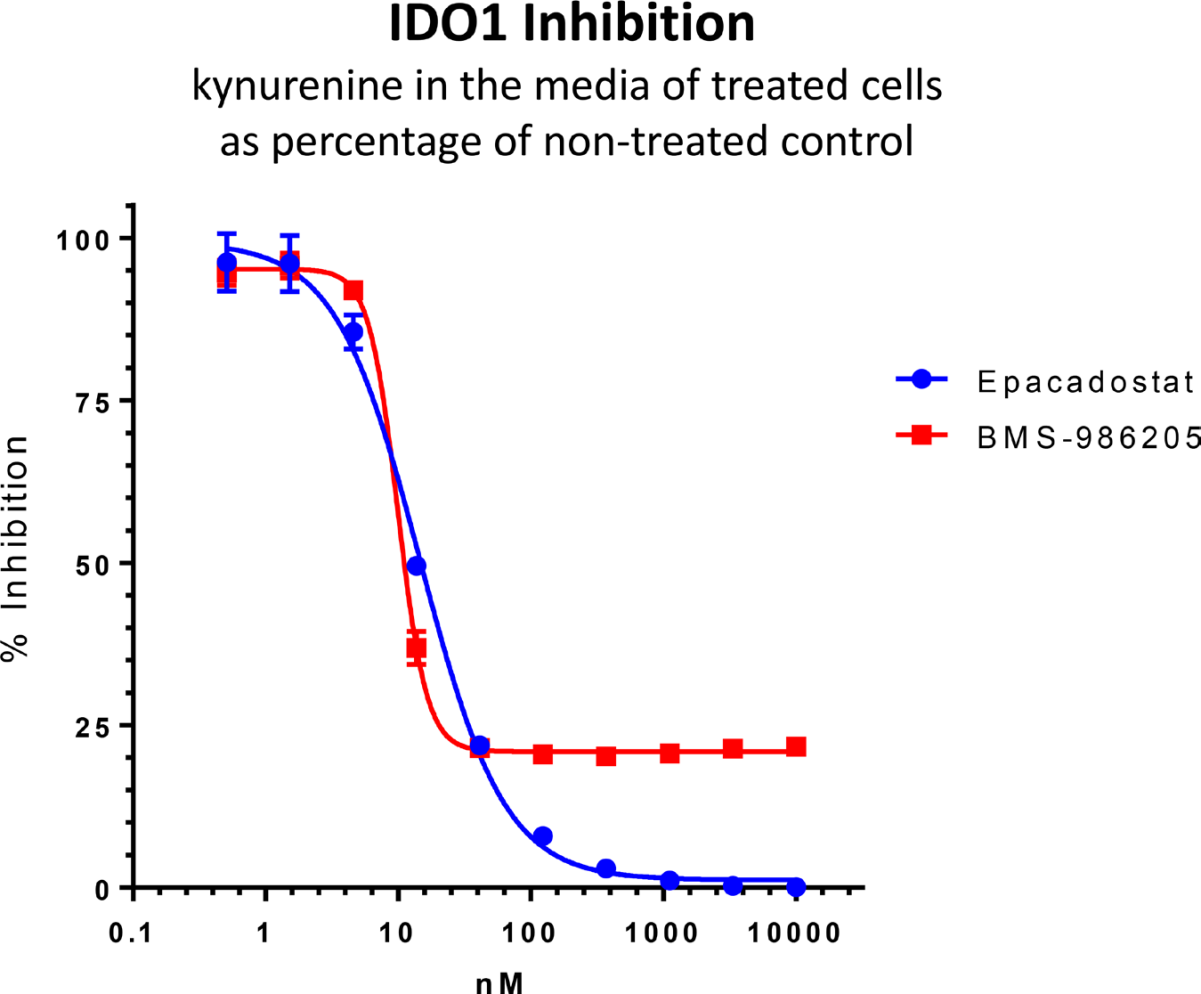
Cell based kynurenine assay After IDO1 induction with IFNγ SKOV-3 cells were treated with epacadostat and BMS-986205. Kynurenine levels were measured in the media and kynurenine concentrations in the wells treated with inhibitors were plotted as a percentage of the non-treated control. Error bars represent standard deviations of three replicates.

Representative assay data for epacadostat and BMS-986205 are shown on Figure 1. IC50 values of the two compounds were in the low nanomolar range – ~15.3 nM for epacadostat and ~9.5 nM for BMS-98620. At low micromolar concentrations epacadostat completely inhibited IDO1 activity while BMS-98620 achieved only 80% maximum inhibition. The slope of BMS-98620 dose response curve was much steeper than that of epacadostat (Hill slope 0.2575 versus 0.07792).

The two compounds inhibit IDO1 via different mechanisms. Epacadostat is a competitive reversible IDO1 inhibitor that binds to the heme cofactor [12, 19, 20]. BMS-986205 is an irreversible IDO1 inhibitor [12], it was recently found to compete with heme binding to apo-IDO1. [21]. Apo form of IDO1 forms after heme dissociation and is predominant in SKOV-3 cells [21]. Since BMS-986205 can only inhibit the apo form its maximum effect is limited by availability of the apo form and rate of conversion of the active form into the apo form. Some substrate can be catalyzed by the active enzyme present in cells before heme dissociation takes place and BMS-986205 has a chance to inhibit these enzyme molecules. Indeed, when we pre-incubated BMS-986205 with cells before adding IDO1 substrate (tryptophan) the maximum effect of IDO1 increased (though did not reach 100%, data not shown). On the other hand, epacadostat can quickly inhibit an active enzyme form by binding to its heme. This explains the difference in the maximum effect we observed with the two compounds.

After media is removed from assay plate(s) for kynurenine analysis the cells in the assay plate(s) can also be analyzed for viability and caspase induction to identify potentially toxic compounds early in the screening process. Cell viability can be assessed right after media removal, or cells can be incubated for an extended time (e.g. 48 h) after replacing media containing serially diluted compound(s). In our experience the majority of toxic compounds induce caspase 3/7 activation within 24 h but the effect on cell viability becomes more pronounced after 48–72 h incubation. However, particularly toxic compounds may reduce cell viability even after a relatively short assay duration of 24 h. Naturally, reduction in cell viability will also lead to reduced conversion of tryptophan to kynurenine in the media. Thus, assessing cell viability will help to eliminate cytotoxic and /or false positive IDO1 inhibitors.

Cell viability was not affected by either epacadostat and BMS-986205, viable cells numbers were equal throughout the test plate.

### Co-culture Jurkat T cell activation assay

Similar to the above described assay SKOV-3 cells were used as a source of IDO1. They were plated in 96-well flat bottom plate(s) at 1 × 10^4^ cells/well in McCoy's 5a media with 10% FBS and allowed to attach overnight. We found this cell density optimal to achieve good assay robustness, reproducibility, dynamic range and sensitivity to compound inhibition. Lower cell density increases assay sensitivity due to lower target concentrations but lowers dynamic range. Likewise, higher cell density leads to lower sensitivity to IDO1 inhibitors (more target present) and higher dynamic range.

The day after cell seeding (once SKOV-3 cells are attached to a plate) IDO1 was induced by addition of 100 ng/mL IFNγ and cell were incubated overnight at 37°C and 5% CO_2_.

The next day media from SKOV-3 assay plate(s) was replaced with RPMI-1640 −10% FBS media containing serially diluted inhibitor compound. Jurkat T cells were then added to the assay plate(s) at 1 × 10^4^ cells per well and stimulated by addition of 1.6 μg/mL phytohemagglutinin (PHA) and 1 μg/mL phorbol 12-myristate 13-acetate (PMA).

The SKOV-3 and Jurkat co-cultures were incubated for 2 days after which Jurkat T cell activation was assessed by analyzing IL-2 secreted into the media by ELISA. IL-2 was undetectable in the media from unstimulated Jurkat T cells. After stimulation and in the absence of IFNγ (without IDO1 induction) we detect approximately 0.8 ng/mL of IL-2 secreted into the media. When IDO1 is induced in SKOV-3 cells by IFNγ IL-2 secretion decreases to about 0.3 ng/mL.

**Figure 2:**
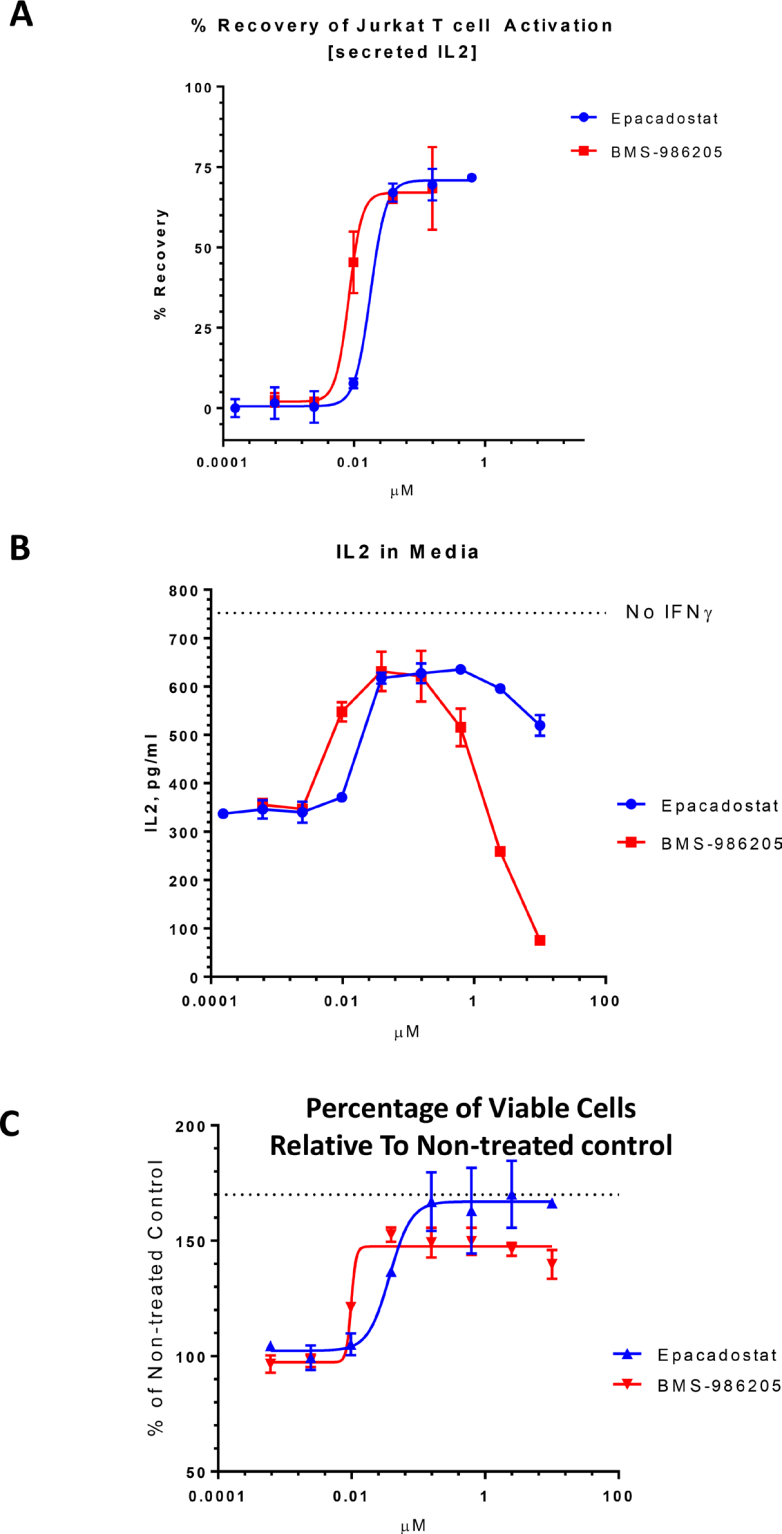
Co-culture functional assay Jurkat T cells were added to IDO1 expressing SKOV-3 cells, stimulated with PHA/PMA and treated with epacadostat and BMS-986205. IL-2 levels in the media were determined by ELISA. (**A**) % Recovery from IDO1 inhibition was calculated based on IL-2 secretion in the absence of inhibitors in IFNγ treated (0% recovery) versus non-treated (100% recovery) SKOV-3 cells. (**B**) IL-2 concentration in the media was plotted against compound concentration. IL-2 level in the absence of compounds and IFNγ is shown with the dotted line (Jurkat T cell activation in the absence of IDO1 inhibition). (**C**) Cell proliferation in the presence of epacadostat and BMS-986205 relative to non-treated control. Dotted line indicates cell proliferation in the absence of IDO1 induction (in the absence of IFNγ) relative to cells treated with IFNγ (IDO1 is induced). Error bars represent standard deviations of three replicates

Representative data for epacadostat and BMS-986205 are shown in the Figure 2. Dose response curves that correspond to concentrations under 100 nM showed that both compounds rescued Jurkat T cell activation (from IDO1 mediated blockage) with low nM IC50 values, ~18 nM for epacadostat and ~8 nM for BMS-986205 (Figure 2A). However, at 2.5 μM, BMS-986205 had no or slightly negative effect on the secreted IL-2 levels compared with the non-treated control. Moreover, at 10 μM, BMS-986205 almost completely inhibited Jurkat T cell activation (Figure 2B). On the other hand, 10 μM epacadostat partially rescued Jurkat activation though somewhat less than at the lower concentration range from 40 nM to 2.5 μM.

We also assessed relative cell viability at the end of the assay by adding Cell-Titer Fluor to the assay plate(s). The readout shows relative number of viable cells, both SKOV-3 and Jurkat, present in the assay wells. Both compounds increased relative cell viability at concentrations greater than their IC50s for rescuing Jurkat T cell activation (Figure 2C). Increase in viable cell numbers is likely due to IDO1 inhibition and availability of the tryptophan to support cell proliferation, as observed by others [13, 22]. In the SKOV-3 kynurenine assay described above incubation with compound(s) is carried out for only one day. SKOV-3 cells proliferate relatively slowly and after one day of culture the presence of IDO1 inhibitor does not noticeably change viable cell numbers. However, when we evaluated two day compound incubation in the SKOV-3 kynurenine assay we did observe an increase in SKOV-3 cell proliferation after IDO1 inhibition.

Micromolar concentrations of BMS-986205 induced increase in combined SKOV-3 and Jurkat viable cell numbers (compared with non-treated control) indicating that the negative effect on Jurkat T cell activation may not have affected their viability at the readout time.

**Figure 3:**
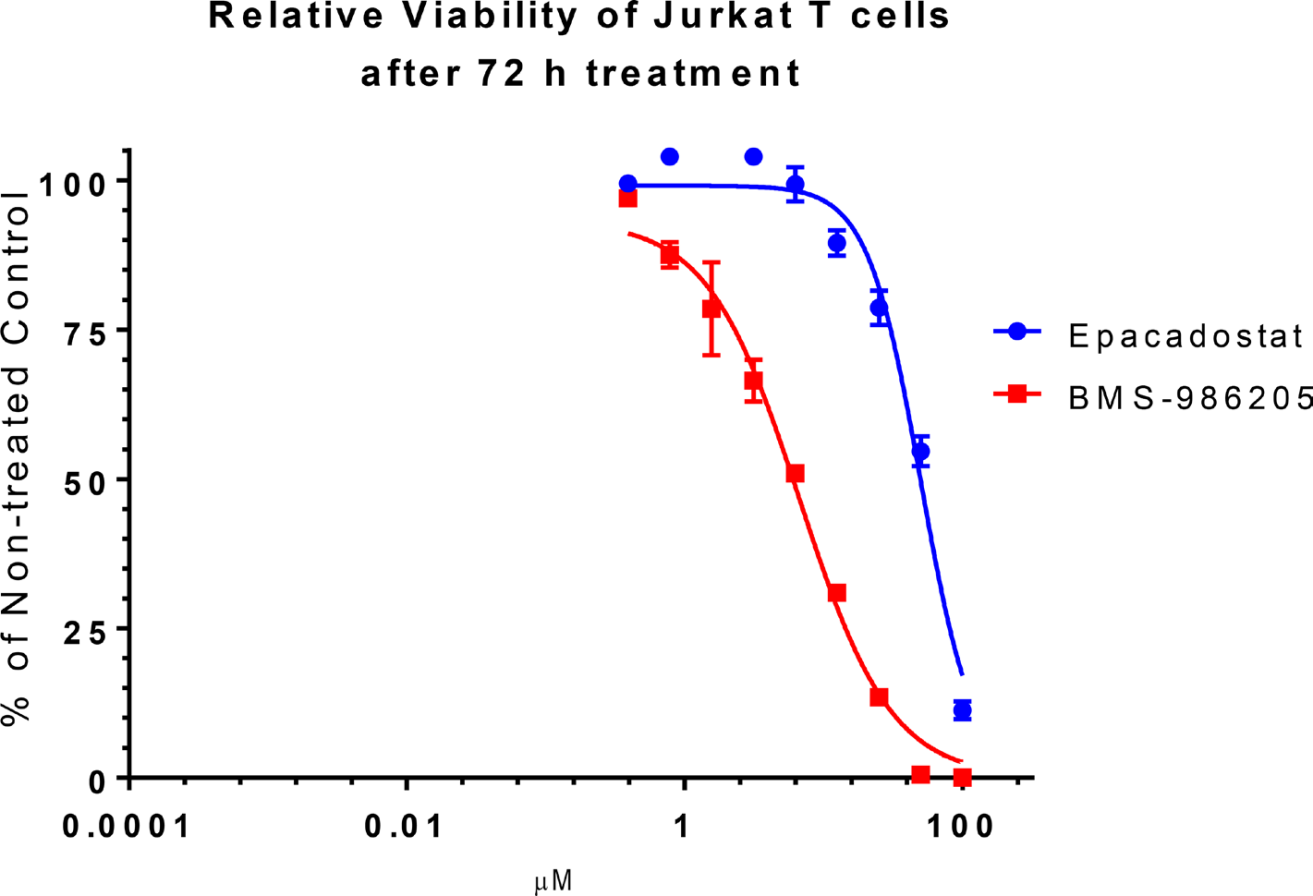
Effect of epacadostat and BMS-986205 on Jurkat cell relative viability Jurkat cells were incubated with serially diluted epacadostat and BMS-986205 for 72 h. Their viability was measured and analyzed as percentage of the non-treated control. Error bars represent standard deviations of three replicates.

We followed up to assess the effect of the two compounds on non-stimulated Jurkat cell viability after 72 h treatment. When present at high micromolar concentrations both compounds reduced the number of viable cells compared with the non-treated control. In case of epacadostat IC50 was 50 μM while BMS-986205 induced cell death at much lower concentrations and its IC50 was 6.3 μM (Figure 3).

The two cells-based assays described above can help researchers evaluate a compound's ability to inhibit intracellular IDO1 and simultaneously assess the presence or absence of other activities such as general cell cytotoxicity that may negatively impact T cell activation. These assays can be used for further characterization of compounds identified in the enzymatic IDO1 assay screen or as a primary screen followed by specificity confirmation in an enzymatic assay. Rohrig *et al*. [23] reported a relatively high failure rate of compounds after the primary IDO1 enzymatic screen mostly due to non-specific IDO1 inhibition mechanisms (e.g. covalent reactivity or redox cycling). Also, enzymatic assay carried at temperatures below 30°C will not detect inhibitors binding to the apo form of IDO1 such as BMS-986205 [21]. We recommend screening compounds at a wide concentration range as some properties may manifest at low and not high (and vice versa) concentrations as we have observed with the two clinical candidates.

In summary, we have developed cell based assays that can be easily implemented and used for screening of IDO1 inhibitors in a physiological context. They can be also useful for screening modalities that alter IDO1 expression or stability. The described assays can facilitate identification and selection of most promising IDO1 targeting molecules for further development. In addition, other targeted agents can be evaluated in these assays for potential effects on IDO1 activity especially when targeting signaling pathways known to modulate IDO1.

## MATERIALS AND METHODS

SKOV-3 and Jurkat clone E6-1 cells were purchased from ATCC and cultured according to manufacturer's instructions.

Trichloroacetic acid, Ehrlich's Reagent (4-(Dimethylamino)benzaldehyde and kynurenine were purchased from Sigma. IFNγ was acquired from ThermoFisher Scientific.

Detection Reagent Solution was prepared by dissolving Ehrlich's Reagent in acetic acid at 20 mg per 1 mL.

Human IL-2 ELISA kit was purchased from BioLegend. 20 μL of each media sample from the functional assay was used for analysis in the ELISA according to the manufacturer's instructions.

The final concentration of DMSO in the cell culture should not exceed 0.3% for the kynurenine assay and 0.2% for the co-culture functional (Jurkat T cell activation) assay.

### SKOV-3 IDO1 assay (cell plating, IDO1 induction, kynurenine determination)

The cells were plated at 3 × 10^4^ cells/well and allowed to attach overnight. The next day IFNγ was added to the cell culture at 100 ng/mL final concentration to induce IDO1 expression followed by 24 h incubation at 37°C and 5% CO_2_.

A kynurenine standard curve was used to interpolate kynurenine concentrations in the test samples.

### Relative viability determination

Cell viability was assessed using Cell-Titer Fluro (Promega) according to manufacturer's instructions. Relative cell viability was calculated based on the reading of the non-treated control. % Relative Viability = TestValue/Non-treatedControlValue × 100.

### Caspase 3/7 activation

Caspase 3/7 activity was measured using Caspase 3/7 Glo reagent form Promega according to manufacturer's instructions.

All experiments were repeated at least three times.
